# MRI is more accurate than FDG-PET in assessing complete response in rectal cancer patients after neoadjuvant therapy

**DOI:** 10.1007/s00423-025-03679-8

**Published:** 2025-03-25

**Authors:** Yehuda Kariv, Ronen Berkovitz, Reut El-On, Alexander Barenboim, Hagit Tulchinsky, Meir Zemel, Oded Brautbar, Dan Mirelman, Sharon Pelles-Avraham, Ravit Geva, Inna Ospovat, Guy Lahat, Jonathan B. Yuval

**Affiliations:** 1 Division of Surgery, Tel Aviv University and Tel Aviv Sourasky Medical Center (Ichilov), 6 Weizmann Street, 6423906 Tel Aviv, Israel; 2 Colorectal Service, Tel Aviv University and Tel Aviv Sourasky Medical Center (Ichilov), 6 Weizmann Street, 6423906 Tel Aviv, Israel; 3 Department of Radiology, Tel Aviv University and Tel Aviv Sourasky Medical Center (Ichilov), 6 Weizmann Street, 6423906 Tel Aviv, Israel; 4 Department of Medical Oncology, Tel Aviv University and Tel Aviv Sourasky Medical Center (Ichilov), 6 Weizmann Street, 6423906 Tel Aviv, Israel; 5 Department of Radiation Oncology, Tel Aviv University and Tel Aviv Sourasky Medical Center (Ichilov), 6 Weizmann Street, 6423906 Tel Aviv, Israel

**Keywords:** Rectal cancer, Positron emission tomography, MRI, Radiology

## Abstract

**Purpose:**

The role of FDG-PET in the restaging rectal cancer following neoadjuvant therapy (NAT) is not clear. We compared the accuracy of FDG-PET and MRI in the assessment of rectal cancer response to NAT.

**Methods:**

Data of patients treated between January 2015 and September 2022 were captured from a rectal tumor registry. Restaging FDG-PET and MRI were evaluated for the presence of viable tumor. Imaging was compared to the reference standard of pathological results for patients that underwent surgery, and sustained clinical complete response for patients that entered watch and wait. Sensitivity was defined as correctly identifying patients with a complete response.

**Results:**

Eighty-two patients met the inclusion criteria. Of these, 60 patients underwent restaging MRI and 54 underwent restaging FDG-PET. Thirty-two were evaluated by both modalities. Mean age and distance from anal verge were 59.9 ± 12.7 years and 5.9 ± 3.2 cm. Baseline staging was cT1-2, cT3 and cT4 for 7 (8.5%), 62 (75.6%) and 13 (15.9%) of the patients, respectively. Baseline nodal staging was cN0 and cN + for 32 (39%) and 50 (61%) of the patients, respectively. All patients were treated with radiation with the majority 73 (89%) receiving chemoradiotherapy. There were 17 patients (21%) that had a pathological or sustained clinical complete response. All baseline characteristics were not meaningfully different between groups. MRI was more accurate than FDG-PET in all parameters including sensitivity, specificity, positive and negative predictive value and overall accuracy.

**Conclusion:**

MRI outperforms FDG-PET in the identification of complete response in rectal cancer patients after NAT.

## Introduction

In about a quarter of patients with locally advanced rectal cancer treated with neoadjuvant chemoradiotherapy, no viable tumor cells remain in the surgical specimen, a finding called pathological complete response (pCR) [1]. Rectal cancer patients with pCR have excellent oncological outcomes [1]. With the addition of 4–6 months of systemic chemotherapy to chemoradiation, in a treatment called total neoadjuvant therapy (TNT) pCR rates can approach 40% [2]. Because of the excellent oncological outcomes of patients with pCR and the sequelae of urinary, bowel and sexual dysfunction caused by radical surgery, some practitioners try and identify complete response clinically before rectal resection and in patients with complete clinical or near complete clinical response, replace radical surgery with strict surveillance, commonly called watch and wait (WW) [3, 4]. The organ preservation in rectal adenocarcinoma (OPRA) trial (NCT02008656), showed that this approach was both safe, with similar disease-free survival to historical controls, and efficacious in terms of rectal preservation, with nearly half (47%) of patients preserving their rectum at 5 years following randomization [5, 6].

Nevertheless, correct clinical identification of true response remains challenging [4, 7]. More than a third of patients entering strict surveillance in WW, experience a local tumor regrowth during follow up [5], demonstrating an inaccuracy of specificity in the clinical assessment of complete response. Additionally, around 15% of patients that undergoing radical surgery due to clinical incomplete response, actually have a pCR in the resected specimen [3, 8], showing inaccuracy of sensitivity in the clinical assessment of complete response. There is still a need to more accurately diagnose true tumor response to avoid unnecessary surgery on the one hand and to avoid leaving patients with viable rectal tumors on the other hand.

Clinical assessment of response to neoadjuvant therapy (NAT) is generally performed by combination of physical examination, endoscopy and rectal MRI. These were the modalities used in the OPRA trial. The accuracy of these modalities is only around 60–70% in recognizing true response [7, 9, 10]. Although FDG-PET is infrequently used for restaging of rectal cancer, our center commonly utilizes FDG-PET at baseline and following NAT to help assess both local disease stage at baseline and local tumor response at restaging and for the presence of distant metastases at both time points. The usage of FDG-PET in this context is partly due to the relative availability of this modality in our country and partly due to clinicians' preference and reliance on this imaging modality. The role of FDG-PET in the restaging of rectal cancer following neoadjuvant therapy is not clear and may help in properly assessing true tumor response to NAT. Our aim was to compare the diagnostic performance of FDG-PET and MRI in identifying complete response among patients with rectal cancer treated with NAT.

## Materials and methods

### Patients

This is a retrospective cohort study. The study population consisted of patients with rectal cancer seen at our center between January 2015 and September 2022 and treated with NAT followed by either restaging FDG-PET or MRI or both and then either surgery or WW (the latter in patients with clinical complete response). WW was performed according to established protocols such as published in the Organ Preservation in Rectal Adenocarcinoma Trial [4, 6]. Patients without restaging imaging, patients that did not receive NAT, patients with rectal tumor other than adenocarcinoma and patients that were recommended surgery but refused were excluded. Data on patient, treatment, and tumor characteristics were collected following approval from the institutional review board, with approved waiver of informed consent (IRB number TLV-0271–23 issued on June 19th 2023). The management of all patients in the study was decided following a multi-disciplinary team meeting comprised of surgeons, medical oncologists, radiation oncologists, radiologists, gastroenterologists and pathologists.

### MRI

MR images were acquired with MRI units with a field strength of 1.5T or 3T. A phased coiled array was used for signal reception. T2 weighted (T2W) images were acquired in the sagittal, coronal and axial planes and diffusion weighted (DW) images were acquired in the axial plane. The axial plane of both T2W and DW images were performed at identical planes perpendicular to the rectum at the site of the tumor (oblique axial plane). Each scan was evaluated by an attending radiologist with at least 5 years as a board-certified radiologist.

### F-18 FDG PET

Whole body imaging from vertex to mid-thigh were obtained by PET/CT units in accordance with accepted protocol of European Association of Nuclear Medicine (EANM). All patients fasted for at least 4 h prior to radiotracer injection. An intravenous dose of F-18 FDG adjusted to the patients' weight (4Mbq/kg) was administered, and the images were acquired approximately one hour later. Patients voided before image acquisition. Reconstruction was performed simultaneously in the sagittal, coronal and axial planes.

### Reference standards

The reference standard for true response was pCR for patients that underwent surgery or clinical complete response (cCR) of > 2 years for patients in WW. The reference standard for incomplete response was cancer cells in the resected specimen for patients that underwent surgery or persistent tumor or tumor regrowth for patients in WW. Complete response was defined as the positive outcome; sensitivity was defined as the proportion of patients with true complete response correctly classified by imaging and specificity was defined as the proportion of patients with true incomplete response correctly identified by imaging.

### Statistical analysis

Categorical variables are presented as frequency and percent. Continuous variables are presented as mean ± standard deviation. Univariate analysis was performed to identify differences between groups utilizing the chi-square test of independence categorical variables and the Student's t-test for continuous variables. Sensitivity, specificity, positive and negative predictive values as well as overall accuracy was calculated for both FDG-PET and MRI. All statistical analyses were performed using SPSS software version 29 (IBM).

## Results

**Fig. 1:**
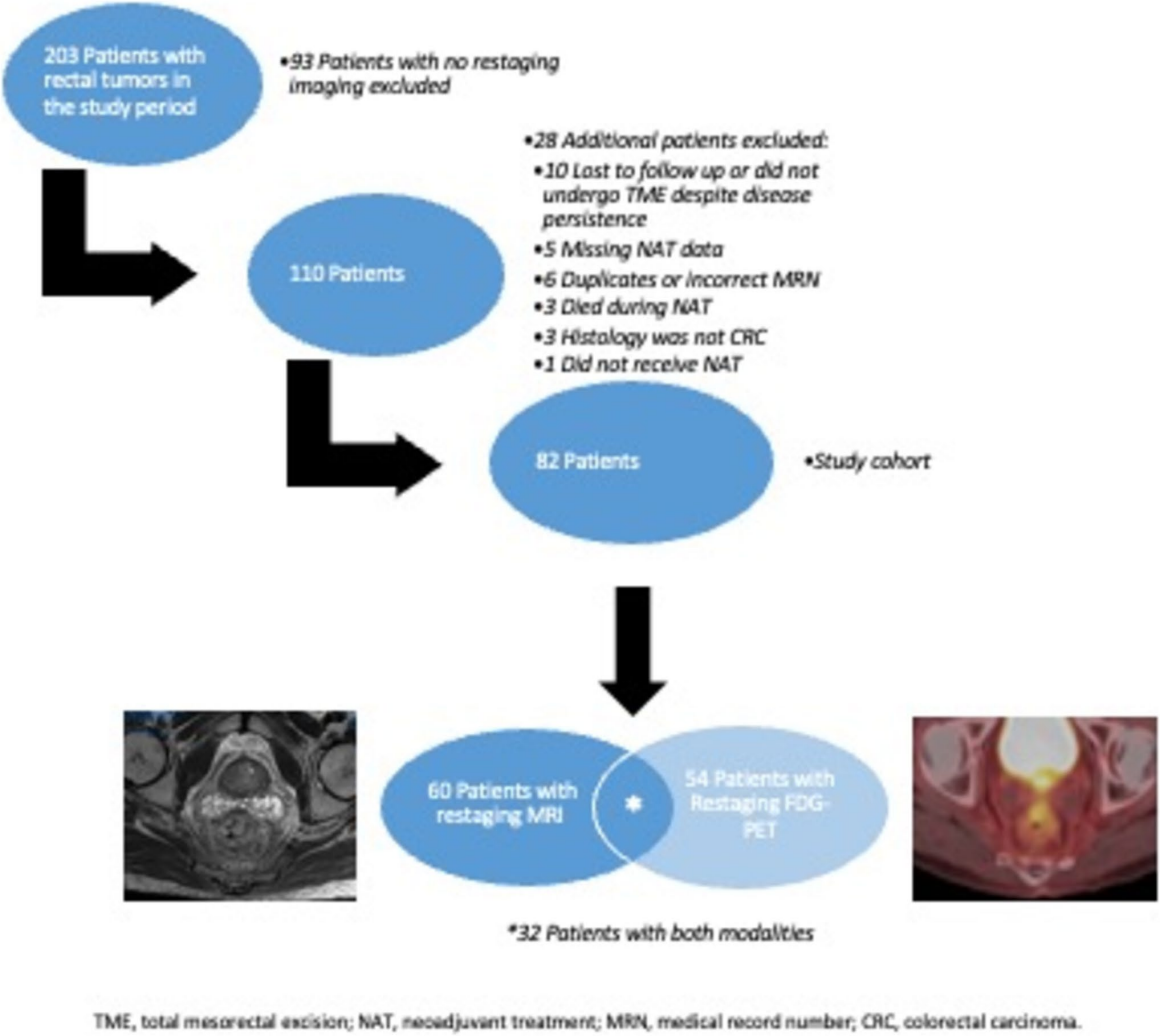
Study flow chart

During the study period 203 patients with rectal tumors were seen at our medical center. Of these, 93 patients were excluded because they did not have any restaging imaging. 28 additional patients were excluded for the following reasons: 10 were lost to follow up or did not undergo surgery despite clinical disease persistence, 5 had missing NAT data, 6 patients were actually duplicates, 3 died during NAT, 3 had histology other than adenocarcinoma, and one did not receive NAT. The remaining 82 patients comprised the study cohort. Of these 60 (73.2%) patients underwent restaging MRI, 54 (65.8%) underwent restaging FDG-PET and 32 (39.0%) underwent restaging by both modalities (Fig. 1).

Baseline patient, tumor and treatment characteristics can be seen in Table 1. Mean age was 59.9 ± 12.7 years and 36 patients (43.9%) were female. Mean height of the tumor from the anal verge was 5.9 ± 3.2 cm. Baseline staging was cT1-2, cT3 and cT4 for 7 (8.5%), 62 (75.6%) and 13 (15.9%) of the patients, respectively. Baseline nodal staging was cN0 and cN + for 32 (39.0%) and 50 (61.0%) of the patients, respectively. Two patients (2.4%) had metastatic disease at diagnosis. All patients received neoadjuvant radiation with most (89.0%) receiving long course chemoradiotherapy (CRT). The majority of patients (70.7%) did not receive neoadjuvant systemic chemotherapy.

**Table 1:** Patient, tumor and treatment characteristics

Characteristic	Number (%) (*N* = 82)
Age*	59.9 ± 12.7 years
Female	36 (44.0%)
Distance from anal verge*	5.9 ± 3.2 cm
cT category	
1/2	7 (8.5%)
3	62 (75.6%)
4	13 (15.9%)
cN category	
Negative	32 (39.0%)
Positive	50 (61.0%)
cM category	
Loco-regional disease	80 (97.6%)
Metastatic disease	2 (2.4%)
Initial approach	
WW	11 (13.4%)
TME	69 (84.1%)
LE	2 (2.4%)
Neoadjuvant radiation therapy	
SCRT	9 (11.0%)
CRT	73 (89.0%)
Neoadjuvant chemotherapy	
CapOX	7 (8.5%)
FOLFOX	14 (17.1%)
Other	3 (3.7%)
None	58 (70.7%)
pCR or sustained cCR	17 (20.7%)

^*^Mean ± standard deviation

*WW* watch and wait, *TME* total mesorectal excision, *LE* local excision, *SCRT* short course radiotherapy, *CRT* chemo-radiotherapy, *CapOX* capecitabine, oxaliplatin, *FOLFOX* leucovorin, fluorouracil, oxaliplatin, *pCR* pathological complete respone, *cCR* clinical complete response

Following NAT, initial treatment approach was WW in 11 patients (13.4%), radical resection in 69 patients (84.1%) and local excision in 2 patients (2.4%). According to the reference standard, 17 (20.7%) patients had a pCR or sustained cCR of more than 2 years.

Mean time from NAT to MRI and FDG-PET was 50.8 ± 59.2 and 46.6 ± 44.6 days, respectively. Mean time from MRI and FDG-PET to surgery was 94.3 ± 134.9 and 94.1 ± 125.0 days, respectively.

There was no meaningful difference in baseline patient, tumor and treatment characteristics between the group of patients that underwent restaging MRI and the group of patients that underwent restaging FDG-PET (Table 2).

**Table 2:** Comparison of Patient, Tumor and Treatment Characteristics between Patients with Restaging MRI and Those with Restaging FDG-PET

Characteristic	MRI Number (%) (*N* = 60)	FDG-PET Number (%) (*N* = 54)	*P* Value
Age*	60.3 ± 13.3 years	60.0 ± 11.5 years	0.904
Female	28 (46.7%)	24 (44.4%)	0.852
Distance from anal verge*	5.8 ± 3.4 cm	6.0 ± 3.2 cm	0.815
cT category			0.986
1/2	4 (6.7%)	4 (7.4%)	
3	44 (73.3%)	39 (72.2%)	
4	12 (20.0%)	11 (20.3%)	
cN category			0.630
Negative	26 (43.3%)	21 (38.9%)	
Positive	34 (56.7%)	33 (61.1%)	
cM category			0.915
Loco-regional disease	58 (96.7%)	52 (96.3%)	
Metastatic disease	2 (3.3%)	2 (3.7%)	
Initial approach			0.947
WW	7 (11.7%)	9 (16.7%)	
TME	51 (85.0%)	43 (79.6%)	
LE	2 (3.3%)	2 (3.7%)	
Neoadjuvant radiation therapy			0.093
SCRT	4 (6.7%)	9 (16.7%)	
CRT	56 (93.3%)	45 (83.3%)	
Neoadjuvant chemotherapy			0.947
CapOX	5 (8.3%)	5 (9.3%)	
FOLFOX	10 (16.7%)	11 (20.4%)	
Other	3 (5.0%)	3 (5.6%)	
None	42 (70.0%)	35 (64.8%)	
pCR or sustained cCR	12 (20.0%)	13 (24.1%)	0.600

^*^Mean ± standard deviation

*WW* watch and wait, *TME* total mesorectal excision, *LE* local excision, *SCRT* short course radiotherapy, *CRT* chemo-radiotherapy, *CapOX* capecitabine, oxaliplatin, *FOLFOX* leucovorin, fluorouracil oxaliplatin, *pCR* pathological complete respone, *cCR* clinical complete response

Accuracy tables comparing restaging MRI interpretation and restaging FDG-PET interpretation with the reference standard can be seen in Table 3 and Table 4, respectively. All accuracy parameters of MRI interpretation were better than those of FDG-PET interpretation. These included overall accuracy (78.3% vs. 68.5%), sensitivity (58.3% vs. 53.8%), specificity (83.3% vs. 73.2%), positive predictive value (PPV) (46.7% vs. 38.9%) and negative predictive value (NPV) (88.9% vs. 83.3%).

**Table 3:** MRI accuracy parameters

	pCR or scCR	Residual Tumor on Pathology or WW	Total
CR on MRI	7	8	15
Residual Tumor on MRI	5	40	45
Total	12	48	60

Sensitivity – 58.3%, Specificity – 83.3%, Positive Predictive Value – 46.7%, Negative Predictive Value – 88.9%, Overall Accuracy – 78.3%

*CR* Complete Response, *pCR* Pathological Complete Response, *scCR* Sustained Clinical Complete Response, *WW* Watch and Wait

**Table 4:** FDG-PET accuracy parameters

	pCR or scCR	Residual Tumor on Pathology or WW	Total
CR on FDG-PET	7	11	18
Residual Tumor on FDG-PET	6	30	36
Total	13	41	54

Sensitivity – 53.8%, Specificity – 73.2%, Positive Predictive Value – 38.9%, Negative Predictive Value – 83.3%, Overall Accuracy – 68.5%

*CR* Complete Response, *pCR* Pathological Complete Response, *scCR* Sustained Clinical Complete Response, *WW* Watch and Wait

In the subset of 32 patients that underwent restaging by both modalities, there was agreement in the interpretation of presence (or absence) of residual tumor in 26 patients (81.3% of this subset) and disagreement in 6 patients (18.8%). Among patients with discrepancy between MRI and FDG-PET, in 3 patients (9.4%) restaging MRI was interpreted as having residual tumor and FDG-PET was interpreted as not having residual tumor and in 3 patients (9.4%) the opposite was true. According to the reference standard 4 of these 6 patients had residual tumor and 2 did not. Among these 6 patients, tumor response was correctly recognized by both MRI and FDG-PET in two of four patients with residual tumor and one of two patients with complete response.

The subset of patients receiving TNT included 24 patients, 13 of which were restaged by both modalities. Of the 24 patients treated with TNT, a total of 18 patients were restaged with MRI and 19 were restaged with FDG-PET. Accuracy parameters for this subset of patients on MRI and FDG-PET were: overall accuracy (77.8% vs. 73.7%), sensitivity (0% vs. 40.0%), specificity (87.5% vs. 85.7%), PPV (0% vs. 50.0%) and NPV (87.5% vs. 80.0%). According to the reference standard, only 2 patients and 5 patients had a complete response in the MRI and FDG-PET groups, respectively.

## Discussion

The findings of our study show that following NAT for rectal cancer patients, comprising most often of CRT without systemic chemotherapy, MRI interpretation is superior to FDG-PET in identifying complete response in all accuracy parameters. In addition, both MRI and FDG-PET showed lower sensitivity than specificity, which demonstrates a bias toward overcalling residual tumor and under-calling complete response. In cases when both MRI and FDG-PET are utilized for restaging in the same patient, their interpretations are largely concordant.

Overall accuracy was 78% for MRI and only 69% for FDG-PET. The accuracy of MRI described herein is consistent with previous data from a meta-analysis of 16 studies comprising of 790 patients [10] that showed an overall accuracy of detecting complete response of 75%. In contrast to previous studies which showed higher specificity than sensitivity [7, 10], in this study both MRI and FDG-PET showed lower sensitivity (58% and 54%) than specificity (83% and 73%) demonstrating that although errors occur in both directions, there is a bias toward over diagnosing residual tumor and under diagnosing complete response. Average time from imaging to surgery in our study was approximately three months for both MRI and FDG-PET. Tumor response may have continued during this time, which may explain, in part, the bias to overcalling residual tumor in both modalities. However, since there was no meaningful difference in the time between the two imaging modalities and surgery, this time period does not explain the difference in overall accuracy between FDG-PET and MRI. The bias towards overcalling residual tumor may also have affected, in part, by the small sample size of 12 and 13 patients with complete response according to the reference standard in the MRI and FDG-PET groups, respectively. In fact, the rate of complete response in the study cohort of only 21% is lower than reported rates of complete response in some studies [2, 5, 6, 11, 12] but not others [13, 14].

The low rate of complete response may reflect in part the rate of utilization of both TNT as the initial treatment and WW as an initial management strategy in 29% and 13% of the patients, respectively. The rates of complete response and TNT use described herein (21% and 29%, respectively), although low in comparison to recent clinical trials [5, 6, 11, 12], seem to reflect real world data and are similar to rates in the National Cancer Database (NCDB) data of 18%, 16%, respectively [14]. To our knowledge the number and proportion of patients treated with WW are not entered into the NCDB and therefore real-world adoption rates of this management strategy in the United States are not clear. A nationwide study of radiotherapy use in the Netherlands showed that 12% of locally advanced rectal cancer patients were treated by long course chemoradiation and WW, which is very similar to the proportion of patients managed by WW in our cohort (13%)[15].

In the guidelines of the national comprehensive cancer network (NCCN) for rectal cancer [16], FDG-PET is not recommended for baseline staging or restaging following NAT. In addition, FDG-PET was not routinely used for the assessment of rectal cancer response following NAT in historical cohorts utilizing WW [3, 8] or in clinical trials employing this approach [5, 6]. Despite these guidelines and historical trends, oncologists in our country frequently utilize FDG-PET for baseline staging, assessment of response and long term follow up of rectal cancer patients. This practice, which may be uncommon worldwide, gave us the opportunity to investigate the yield of this imaging modality in the assessment of complete response of rectal cancer to NAT. Although rarely used, there are some previous studies investigating the role of FDG-PET in assessment of rectal cancer response to NAT. In a prospective study of 121 patients with rectal cancer receiving neoadjuvant chemoradiation following by radical surgery, Guillem and colleagues investigated the accuracy of restaging FDG-PET and CT in comparison to pathology of the resected specimen [13]. 26 patients (21%) had a pCR on pathology. The authors found the following accuracy parameters: overall accuracy 64%, sensitivity 54%, specificity 66%, PPV 30% and NPV 84%. It is important to note that no patients were treated with TNT and no patients were managed by WW. The FDG-PET related accuracy parameters shown by the authors are similar to ours both in terms of overall accuracy (64% vs. 69%) and higher specificity than sensitivity. In a small prospective trial, Denecke and colleagues investigated the interpretation of response of CT, MRI and FDG-PET in 23 patients with locally advanced rectal cancer that underwent NAT including CRT and local hyperthermia followed by TME [17]. In this trial, which preceded wide adoption of WW, response was categorized as some-response versus no-response and pathology was used as the reference standard. Among the three modalities, FDG-PET showed the highest sensitivity in recognizing some-response (100%) but the lowest specificity in recognizing no-response (60%). Differences in findings from our study are most likely due to the varying categorization/definition of response groups, i.e. some-response vs. no-response as opposed to complete response vs. incomplete response.

Accurate assessment of rectal cancer response to NAT is complex. It is increasingly recognized that the degree of response of rectal cancer to NAT and to radiation in particular is time-dependent [2, 18]. In fact, many tumors that show a meaningful but not a complete response, with time respond completely. These patients are known as near-complete responders, and many advocate a trial of WW in this group as long as the tumor continues to decrease in size. Because the response of the tumor may evolve over time, accuracy parameters are dependent on the timing of the imaging used for response assessment in relation to NAT administration and on the length of time between restaging and surgery. Theoretically there could be residual tumor correctly interpreted on imaging at time point A, which disappears by the time surgery is performed at time point B. In addition, the timing of surgery can affect the pCR rate [2, 19, 20], potentially changing the outcomes of the reference standard by which the accuracy of the imaging is determined. Adding to the complexity, imaging-based evaluation of rectal cancer response to NAT is associated with substantial variability of interpretation between radiologists [7].

Management strategies and technological innovations may help in improving imaging-based response assessment of rectal cancer to NAT. Adding physical examination, and endoscopy findings to the imaging interpretations as well as assessment at more than one time point (and by more than one modality) have been suggested as ways to improve accuracy [7]. In fact, in the OPRA trial, patients with complete or near complete response were assessed by three modalities over a period of time and errors of sensitivity were quite low with a rate of pCR of only 8.5% in patients undergoing radical surgery for clinical suspicion of incomplete response [6]. Errors of specificity were higher with 36% of patients that entered WW, experiencing regrowth during a median follow up of 5.1 years [5]. However, the clinical significance of these errors was low since these regrowths were treatable by curable intent by radical surgery in the vast majority of cases. Some technological innovations may also help clinicians assess rectal cancer response to NAT more accurately. For example, artificial intelligence (AI) utilizing information recorded by MRI and FDG-PET that is not visually represented, also known as radiomics, has been shown to be superior to clinical assessment by expert radiologists [21]. Another new and interesting technology that may help in the correct assessment of tumor response and selection of patients for WW is circulating tumor DNA (ctDNA). A recent prospective trial demonstrated increased diagnostic accuracy of rectal cancer response assessment following NAT when ctDNA data is analyzed alongside the MRI interpretation [22]. Further investigation in large clinical trials of these technologies and management strategies is necessary. However, our study supports that FDG-PET may not be helpful as a technological adjunct to the assessment of complete response of rectal cancer to NAT.

Our study has several limitations, which include selection bias and underreporting inherent to the retrospective design. Furthermore, the study was conducted at a single center which may limit generalizability. Each MRI and FDG-PET was interpreted by a single reader and therefore we lack data on inter-observer agreement. Although there were no meaningful differences between the two study groups, the time intervals from NAT to imaging and from imaging to surgery varied widely and may have impacted some of the results, such as higher specificity of the imaging modalities. Notwithstanding these limitations, our study provides good evidence that MRI outperforms FDG-PET in the identification of complete tumor response following NAT in rectal cancer patients.

## Conclusion

To summarize, MRI is better than FDG-PET in identifying complete response in rectal cancer patients following NAT. In cases when both modalities are utilized in the same patient, their interpretations are largely concordant. Both these findings question the necessity of FDG-PET as a tool for restaging rectal cancer following NAT. At our center, where adoption of both TNT and WW was low during the study period, the interpretation of both MRI and FDG-PET was biased towards overcalling residual tumor and under-calling complete tumor response to NAT. Many promising technological methods have the potential of improving the care of rectal cancer patients by more accurate assessment of rectal cancer response to NAT and the adoption of a WW strategy for appropriate patients. These methods such as ctDNA and AI integration of radiomics should be evaluated in future large and prospective studies.

## Data Availability

Data will be made available by the corresponding author upon request.
